# Epidemiology and outcome of high-surgical-risk patients admitted to an intensive care unit in Brazil

**DOI:** 10.5935/0103-507X.20200005

**Published:** 2020

**Authors:** João Manoel Silva Júnior, Renato Carneiro de Freitas Chaves, Thiago Domingos Corrêa, Murillo Santucci Cesar de Assunção, Henrique Tadashi Katayama, Fabio Eduardo Bosso, Cristina Prata Amendola, Ary Serpa Neto, Luiz Marcelo Sá Malbouisson, Neymar Elias de Oliveira, Viviane Cordeiro Veiga, Salomón Soriano Ordinola Rojas, Natalia Fioravante Postalli, Thais Kawagoe Alvarisa, Bruno Melo Nobrega de Lucena, Raphael Augusto Gomes de Oliveira, Luciana Coelho Sanches, Ulysses Vasconcellos de Andrade e Silva, Antonio Paulo Nassar Junior, Álvaro Réa-Neto, Alexandre Amaral, José Mário Teles, Flávio Geraldo Rezende de Freitas, Antônio Tonete Bafi, Eduardo Souza Pacheco, Fernando José Ramos, José Mauro Vieira Júnior, Maria Augusta Santos Rahe Pereira, Fábio Sartori Schwerz, Giovanna Padoa de Menezes, Danielle Dourado Magalhães, Cristine Pilati Pileggi Castro, Sabrina Frighetto Henrich, Diogo Oliveira Toledo, Bruna Fernanda Camargo Silva Parra, Fernando Suparregui Dias, Luiza Zerman, Fernanda Formolo, Marciano de Sousa Nobrega, Claudio Piras, Stéphanie de Barros Piras, Rodrigo Conti, Paulo Lisboa Bittencourt, Ricardo Azevedo Cruz D’Oliveira, André Ricardo de Oliveira Estrela, Mirella Cristine de Oliveira, Fernanda Baeumle Reese, Jarbas da Silva Motta Júnior, Bruna Martins Dzivielevski da Câmara, Paula Geraldes David-João, Luana Alves Tannous, Viviane Bernardes de Oliveira Chaiben, Lorena Macedo Araújo Miranda, José Arthur dos Santos Brasil, Rafael Alexandre de Oliveira Deucher, Marcos Henrique Borges Ferreira, Denner Luiz Vilela, Guilherme Cincinato de Almeida, Wagner Luis Nedel, Matheus Golenia dos Passos, Luiz Gustavo Marin, Wilson de Oliveira Filho, Raoni Machado Coutinho, Michele Cristina Lima de Oliveira, Gilberto Friedman, André Meregalli, Jorge Amilton Höher, Afonso José Celente Soares, Suzana Margareth Ajeje Lobo

**Affiliations:** 1 Hospital Israelita Albert Einstein - São Paulo (SP), Brazil.; 2 Hospital das Clínicas, Faculdade de Medicina, Universidade de São Paulo - São Paulo (SP), Brazil.; 3 Irmandade da Santa Casa de Misericórdia de Santos - Santos (SP), Brazil.; 4 Hospital do Servidor Público Estadual “Francisco Morato de Oliveira” - São Paulo (SP), Brazil.; 5 Universidade Cidade de São Paulo - São Paulo (SP), Brazil.; 6 Hospital de Câncer de Barretos, Fundação Pio XII - Barretos (SP), Brazil.; 7 Hospital de Base, Faculdade de Medicina de São José do Rio Preto - São José do Rio Preto (SP), Brazil.; 8 Hospital Beneficência Portuguesa - São Paulo (SP), Brazil.; 9 AC Camargo Cancer Center - São Paulo (SP), Brazil.; 10 Hospital Vita Batel - Curitiba (PR), Brazil.; 11 Hospital de Urgências de Goiânia - Goiânia (GO), Brazil.; 12 Hospital Sepaco - São Paulo (SP), Brazil.; 13 Hospital Sírio-Libanês - São Paulo (SP), Brazil.; 14 Associação Beneficente de Campo Grande - Campo Grande (MS), Brazil.; 15 Hospital São Vicente de Paulo - Passo Fundo (RS), Brazil.; 16 Hospital e Maternidade São Luiz Itaim, Rede D’Or - São Paulo (SP), Brazil.; 17 Hospital Pompeia - Caxias do Sul (RS), Brazil.; 18 Hospital das Clínicas, Universidade Federal de Goiás - Goiânia (GO), Brazil.; 19 Vitoria Apart Hospital - Serra (ES), Brazil.; 20 Hospital Português da Bahia - Salvador (BA), Brazil.; 21 Hospital do Trabalhador de Curitiba - Curitiba (PR), Brazil.; 22 Hospital Marcelino Champagnat - Curitiba (PR), Brazil.; 23 Hospital Universitário Cajuru - Curitiba (PR), Brazil.; 24 Hospital Erasto Gaertner - Curitiba (PR), Brazil.; 25 Instituto de Neurologia de Curitiba - Curitiba (PR), Brazil.; 26 Hospital Nossa Senhora de Fátima - Patos de Minas (MG), Brazil.; 27 Hospital Nossa Senhora da Conceição - Porto Alegre (RS), Brazil.; 28 Hospital e Pronto Socorro 28 de Agosto - Manaus (AM), Brazil.; 29 Irmandade da Santa Casa de Misericórdia de Porto Alegre - Porto Alegre (RS), Brazil.; 30 Hospital de Força Aérea do Galeão - Rio de Janeiro (RJ), Brazil.

**Keywords:** Surgical procedures, operative/epidemiology, Surgical procedures, operative/mortality, Postoperative care, Postoperative complications/mortality, Intensive care units, Brazil, Procedimentos cirúrgicos operatórios/ epidemiologia, Procedimentos cirúrgicos operatórios/mortalidade, Cuidados pós-operatórios, Complicações pós-operatórias/mortalidade, Unidades de terapia intensiva, Brasil

## Abstract

**Objective:**

To define the epidemiological profile and the main determinants of morbidity and mortality in noncardiac high surgical risk patients in Brazil.

**Methods:**

This was a prospective, observational and multicenter study. All noncardiac surgical patients admitted to intensive care units, i.e., those considered high risk, within a 1-month period were evaluated and monitored daily for a maximum of 7 days in the intensive care unit to determine complications. The 28-day postoperative, intensive care unit and hospital mortality rates were evaluated.

**Results:**

Twenty-nine intensive care units participated in the study. Surgeries were performed in 25,500 patients, of whom 904 (3.5%) were high-risk (95% confidence interval - 95%CI 3.3% - 3.8%) and were included in the study. Of the participating patients, 48.3% were from private intensive care units, and 51.7% were from public intensive care units. The length of stay in the intensive care unit was 2.0 (1.0 - 4.0) days, and the length of hospital stay was 9.5 (5.4 - 18.6) days. The complication rate was 29.9% (95%CI 26.4 - 33.7), and the 28-day postoperative mortality rate was 9.6% (95%CI 7.4 - 12.1). The independent risk factors for complications were the Simplified Acute Physiology Score 3 (SAPS 3; odds ratio - OR = 1.02; 95%CI 1.01 - 1.03) and Sequential Organ Failure Assessment Score (SOFA) on admission to the intensive care unit (OR = 1.17; 95%CI 1.09 - 1.25), surgical time (OR = 1.001, 95%CI 1.000 - 1.002) and emergency surgeries (OR = 1.93, 95%CI, 1.10 - 3.38). In addition, there were associations with 28-day mortality (OR = 1.032; 95%CI 1.011 - 1.052), SAPS 3 (OR = 1.041; 95%CI 1.107 - 1.279), SOFA (OR = 1.175, 95%CI 1.069 - 1.292) and emergency surgeries (OR = 2.509; 95%CI 1.040 - 6.051).

**Conclusion:**

Higher prognostic scores, elderly patients, longer surgical times and emergency surgeries were strongly associated with higher 28-day mortality and more complications during the intensive care unit stay.

## Abstract (pt)

**Objetivo:**

Definir o perfil epidemiológico e os principais determinantes de morbimortalidade dos pacientes cirúrgicos não cardíacos de alto risco no Brasil.

**Métodos:**

Estudo prospectivo, observacional e multicêntrico. Todos os pacientes cirúrgicos não cardíacos admitidos nas unidades de terapia intensiva, ou seja, considerados de alto risco, no período de 1 mês, foram avaliados e acompanhados diariamente por, no máximo, 7 dias na unidade de terapia intensiva, para determinação de complicações. As taxas de mortalidade em 28 dias de pós-operatório, na unidade de terapia intensiva e hospitalar foram avaliadas.

**Resultados:**

Participaram 29 unidades de terapia intensiva onde foram realizadas cirurgias em 25.500 pacientes, dos quais 904 (3,5%) de alto risco (intervalo de confiança de 95% - IC95% 3,3% - 3,8%), tendo sido incluídos no estudo. Dos pacientes envolvidos, 48,3% eram de unidades de terapia intensiva privadas e 51,7% de públicas. O tempo de internação na unidade de terapia intensiva foi de 2,0 (1,0 - 4,0) dias e hospitalar de 9,5 (5,4 - 18,6) dias. As taxas de complicações foram 29,9% (IC95% 26,4 - 33,7) e mortalidade em 28 dias pós-cirurgia 9,6% (IC95% 7,4 - 12,1). Os fatores independentes de risco para complicações foram Simplified Acute Physiology Score 3 (SAPS 3; razão de chance − RC = 1,02; IC95% 1,01 - 1,03) e Sequential Organ Failure Assessment Score (SOFA) da admissão na unidade de terapia intensiva (RC =1,17; IC95% 1,09 - 1,25), tempo de cirurgia (RC = 1,001; IC95% 1,000 - 1,002) e cirurgias de emergências (RC = 1,93; IC95% 1,10 - 3,38). Em adição, foram associados com mortalidade em 28 dias idade (RC = 1,032; IC95% 1,011 - 1,052) SAPS 3 (RC = 1,041; IC95% 1,107 - 1,279), SOFA (RC = 1,175; IC95% 1,069 - 1,292) e cirurgias emergenciais (RC = 2,509; IC95% 1,040 - 6,051).

**Conclusão:**

Pacientes com escores prognósticos mais elevados, idosos, tempo cirúrgico e cirurgias emergenciais estiveram fortemente associados a maior mortalidade em 28 dias e mais complicações durante permanência em unidade de terapia intensiva.

## INTRODUCTION

The mortality rate and the rate of perioperative complications reported for all surgical patients are 7.7% and 20%, respectively.^(1,2)^ In patients older than 55 years of age undergoing elective surgery, the mortality rate is approximately 8.2%, and complications occur in 15.8% of cases.^(3)^ In cancer patients, the mortality rate is 20.3%, which is significantly higher in emergency surgeries (49.4%) than in elective surgeries (5.7%).^(4)^ A study involving 105,000 surgical patients showed that the presence of any complication in the first 30 days after surgery was the main determinant of the risk of death.^(5)^

In 2011, a study conducted in 28 European countries with 46,539 patients undergoing noncardiac surgery showed a hospital mortality rate of 4%, with significant variation in mortality rates among the various European countries.^(6)^ In Brazil, according to data from the Department of Informatics of the Unified Health System (DATASUS - *Departamento de Informática do Sistema Único de Saúde*), of a total of 4,405,782 surgical procedures performed in 2014, 558,988 (12.7%) were highly complex and had a mortality rate of 2.8%.^(7)^ In addition, a Brazilian study conducted in 21 intensive care units (ICUs) in 2008 showed a 15% ICU mortality rate and a 20.3% 90-day mortality rate in surgical patients, with sepsis (24.7%) being the most common complication observed during the postoperative period.^(8)^

It is known that the clinical outcome of high-risk surgical patients is predominantly influenced by the preoperative physiological state, surgical risk and postoperative care.^(9)^ Thus, updated and more comprehensive data, as well as predictors of the risk of morbidity and mortality of surgical patients in Brazil, are essential.

The objective of this study was to determine the demographic characteristics of surgical patients admitted to Brazilian ICUs, the incidence of and possible factors associated with major postoperative complications, and the 28-day, ICU and hospital mortality rates.

## METHODS

This was a prospective, multicenter cohort study conducted between May 1 and November 1, 2017, with a 28-day follow-up. This study was approved by the Research Ethics Committee of the study’s coordinating center, the *Hospital Israelita Albert Einstein* (CAAE: 55828016.1.1001.0071), and all participating centers. An informed consent form was signed by all patients or their legal guardians. Two participating centers were exempted from the requirement to sign a consent form due to the observational nature of the study.

Recruitment of the participating ICUs was performed in conjunction with the *Associação de Medicina Intensiva Brasileira* network (AMIBnet) through invitations via websites, e-mails and letters individually addressed to each of the intensive care physicians coordinating ICU teams in Brazil. Participants were selected until a sample size with the same proportions as the 2016 census was reached,^(10)^ that is, until the sample comprised approximately 55% of patients from the Southeast, 15% from the South, 15% from the Northeast and 15% from the Central-West and North regions.

Before the beginning of the study, a questionnaire on the structural and operational characteristics of the participating hospitals was sent to the centers that agreed to participate. The intensive care units invited needed to be located in tertiary hospitals with at least one hundred beds, of which at least ten ICU beds were reserved for surgical patients and at least 50% of the patients treated each month were surgical patients. Considering that the higher the number of patients treated was, the better the performance of the included center was,^(11)^ the selected institutions were large hospitals with capacity for and experience in the care of surgical patients who require intensive care during the postoperative period.

Patients aged ≥ 18 years who underwent noncardiac surgery requiring postoperative care in the ICU were included. Because the criteria for postoperative intensive care were not standardized among the centers, all patients with this indication were considered to be high-risk.

Patients with terminal cancer, those receiving palliative care and those with severe liver failure (Child C) were excluded because their inclusion could lead to unrealistic results given that they had little or no prospect of cure. Pregnant women were also excluded.

Furthermore, patients with a hospital stay of less than 12 hours were excluded because it was not possible to determine ICU follow-up or because such stays did not characterize high risk. Patients with multiple reoperations during the same hospital stay and those readmitted to the ICU during the same hospital stay that was considered for inclusion in the study were also excluded because they could not participate more than once in the study.

The data collected included demographic data, Simplified Acute Physiology Score 3 (SAPS 3),^(12)^ Sequential Organ Failure Assessment (SOFA) score on ICU admission,^(13)^ American Society of Anesthesiologists (ASA) physical status classification,^(14)^ comorbidities and characteristics of prioritized surgeries, location of surgery and surgical time. During the first 7 postoperative days or until ICU discharge, whichever came first, the SOFA score^(13)^ and the occurrence of complications were evaluated daily. In addition, ICU and hospital stay times as well as 28-day, ICU and hospital mortality rates were collected. All data were obtained using an electronic form (Research Electronic Data Capture - REDCap).^(15,16)^ Instructions on how to properly complete the data collection form were made available to the researchers.

### Outcomes

The primary outcome was 28-day postoperative mortality, which was evaluated face-to-face or by telephone. A 28-day follow-up period was chosen to standardize the follow-up time related specifically to the surgery.

As secondary outcomes, we assessed the lengths of stay in the ICU and in the hospital, the ICU and hospital mortality, and the incidence of the following complications:

Cardiovascular: characterized by the need for vasopressors for more than 1 hour despite adequate volume resuscitation; acute myocardial infarction; arrhythmias; or cardiac arrest.

Respiratory: a relationship between partial pressure of oxygen and fraction of inspired oxygen (PaO_2_/FiO_2_) < 200 in patients without previous heart disease; the need for reintubation; or the presence of bronchospasm or pneumothorax.

Renal: presence of acute kidney injury determined by an acute increase in serum creatinine by 30% of the baseline value, urine output < 0.5mL/kg/hour, renal SOFA score greater than two points, or the need for renal replacement therapy during the ICU stay in patients with no history of chronic renal failure.

Neurological: Richmond Agitation and Sedation Scale (RASS) ^(17)^ score that acutely fluctuates and is nonzero within 24 hours, agitation as determined by RASS ≥ +2, documented convulsive seizures or stroke.

Coagulation: reduction of platelet count greater than 30% of the baseline value during the preoperative period, platelet count below 100,000mm^3^, or acute bleeding above 100 mL/hour associated with a decrease of 3 hematocrit points.

Gastrointestinal: presence of acute abdominal distension, uncontrolled nausea and vomiting, need for parenteral nutrition, more than three episodes of diarrhea within 24 hours, acute gastrointestinal bleeding, acute liver failure, acute pancreatitis or presence of moderate- to high-output fistulas.

### Statistical analysis

Considering data from the literature, we assumed a minimum mortality rate of 15% in high-risk surgical patients.^(8,18-22)^ We estimated that at least one thousand patients would be necessary for the study, allowing the inclusion of ten explanatory variables in a robust logistic regression model with 28-day mortality as dependent variable.

Categorical variables are presented as absolute and relative frequencies. Quantitative variables are expressed as the mean and standard deviation (SD) or as the median and interquartile range (IQR), as appropriate. We used the Kolmogorov-Smirnov test to evaluate the distribution pattern of continuous numerical variables.

Proportions were compared using the chi-square test or Fisher’s exact test, as appropriate. Quantitative variables were compared with analysis of variance (ANOVA) or the Kruskal-Wallis test, as appropriate.

The associations between explanatory and response variables were evaluated using fixed logistic regression models. Variables that were statistically significant in the univariate analyses (p < 0.05) were selected for the multiple logistic regression models. Collinearity was first evaluated by examining the covariance matrix and Pearson’s correlation coefficient for continuous variables or by cross-tabulation for categorical variables. We also evaluated the collinearity with the analysis of the variance inflation factor. Variables with substantial collinearity (variance inflation factor ≥ 10) were excluded. The results of the logistic regression analyses were expressed as odds ratios (ORs) and their 95% confidence intervals (95%CI).

All probabilities of statistical significance (p-values) were two-tailed. The p-values were considered statistically significant when they were < 0.05. The software Statistical Package for Social Sciences (SPSS Inc.®; Chicago, IL, USA), version 20.0, and R v.3.4.1 (R Foundation for Statistical Computing, Vienna, Austria) were used to perform the analyses.

## RESULTS

### Characteristics of the centers and patients studied

**Figure 1:**
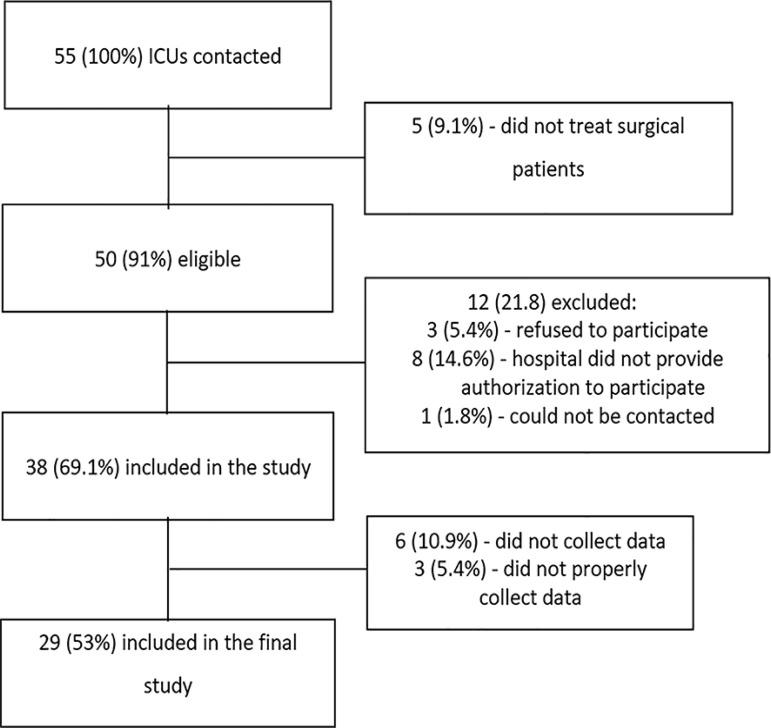
Flowchart of the study participants. ICU - intensive care unit.

A total of 55 ICUs at 55 hospitals were selected for participation in the study. Of these, 12 (21.8%) did not meet the eligibility criteria required for participation for different reasons: 5 ICUs (9.1%) refused to participate because they did not treat a sufficient number of surgical patients, and 9 (16.4%) returned incomplete questionnaires that were missing important data for the study. In total, 29 ICUs participated in the study (Figure 1). Approximately half of the participating ICUs were located in the Southeast Region (14/29; 48.3%), followed by the South (8/29; 27.6%), the Central-West (4/29; 13.7%), and the North and Northeast (3/29; 10.3%) (Table 1). There were no significant differences in the operational characteristics of the ICUs among the regions of the country (Table S1 - Supplementary material).

**Table 1:** Profile of patients included in the study according to geographic distribution

Features	All	Southeast	South	Central-West	North and Northeast	p value
Age	62 (50 - 72)	62 (51 - 72)	63 (49 - 74.5)	57 (39.2 - 70.7)	64 (57-73)	0.225 *
Male sex	444 (53.8)	269 (54.7)	102 (47.4)	57 (68.7)	16 (45.7)	0.008 †
SAPS 3 score	42 (32 - 53)	39 (31 - 49)	44 (34 - 54)	59.5 (43 - 70)	44.5 (37 - 55)	< 0.001 *
SOFA on ICU admission	2 (1 - 5)	2 (1 - 5)	2 (1 - 4)	4 (1 - 7)	1 (1 - 2)	< 0.001 *
BMI	25 (22 - 28)	25 (23 - 28)	25 (23 - 28)	24 (20 - 25)	25 (20 - 27)	< 0.001 *
Ethnicity						
Caucasian	585 (71.3)	343 (71.3)	198 (91.7)	39 (47.0)	5 (12.5)	< 0.001 †
Brown	157 (19.1)	77 (16.0)	13 (6.0)	35 (42.2)	32 (80.0)	< 0.001 †
Black	61 (7.4)	49 (10.2)	1 (0.5)	8 (9.6)	3 (7.5)	< 0.001 †
Other	17 (2.1)	12 (2.4)	4 (1.9)	1 (1.2)	0 (0.0)	0.774 †
ASA	2 (2 - 3)	2 (2 - 3)	2 (2 - 3)	3 (2 - 3)	2 (2 - 3)	0.044 *
Duration of surgery, minutes	240 (180 - 360)	300 (180 - 390)	180 (120 - 300)	180 (120 - 300)	210 (155 - 300)	< 0.001 *
Type of surgery						
Elective	613 (69.2)	401 (76.4)	154 (64.7)	26 (31.3)	32 (80.0)	< 0.001 †
Urgent	147 (16.6)	84 (16.0)	32 (13.4)	26 (31.3)	5 (12.5)	0.002 †
Emergency	126 (14.2)	40 (7.6)	52 (21.8)	31 (37.3)	3 (7.5)	< 0.001 †
Surgeries						
Abdominal	252 (28.1)	130 (24.4)	53 (22.0)	32 (38.6)	37 (92.5)	< 0.001†
Cancer	250 (27.9)	197 (37.0)	27 (11.2)	2 (2.4)	24 (60)	< 0.001 †
Neurological	186 (20.8)	88 (16.5)	79 (32.7)	19 (23.0)	0 (0.0)	< 0.001 †
Orthopedic	143 (16.0)	71 (13.3)	53 (22.0)	19 (23.0)	0 (0.0)	< 0.001 †
Vascular	74 (8.3)	57 (10.7)	8 (3.3)	9 (12.2)	0 (0.0)	< 0.001 †
Thoracic	53 (5.9)	27 (5.1)	16 (6.6)	7 (8.4)	3 (7.5)	0.567 †
Urological	48 (5.4)	40 (7.5)	5 (2.1)	2 (2.4)	1 (2.5)	0.007 †
Head and neck	39 (4.4)	28 (5.3)	9 (3.7)	2 (2.4)	0 (0.0)	0.278 †
Gynecological	19 (2.1)	14 (2.6)	3 (1.2)	1 (1.2)	1 (2.5)	0.589 †
Other surgeries	55 (6.1)	48 (9.0)	4 (1.7)	1 (1.2)	2 (5.0)	< 0.001 †
Underlying disease	707 (80.4)	444 (84.9)	180 (77.3)	52 (62.7)	31 (77.5)	< 0.001 †
Hypertension	396 (44.2)	260 (48.9)	89 (36.9)	28 (33.7)	19 (47.5)	0.003 †
Cancer	191 (21.3)	143 (26.9)	29 (12.0)	4 (4.8)	15 (37.5)	< 0.001 †
Diabetes mellitus	188 (21.0)	131 (24.6)	31 (12.9)	16 (19.3)	10 (25.0)	0.002 †
Smoking	134 (15.0)	85 (16.0)	33 (13.7)	14 (16.9)	2 (5.0)	0.251 †
CI	67 (7.5)	47 (10.6)	8 (3.3)	4 (4.8)	8 (20.0)	< 0.001 †
COPD	54 (6.0)	34 (6.4)	13 (5.4)	3 (3.6)	4 (10.0)	0.520 †
CRF	48 (5.4)	40 (7.5)	2 (0.8)	4 (4.9)	2 (5.0)	0.002 †
Stroke	27 (3.0)	16 (3.0)	8 (3.3)	3 (3.6)	0 (0.0)	0.700 †
Alcoholism	46 (5.1)	28 (5.3)	11/(4.6)	7 (8.4)	0 (0.0)	0.241 †
Arrhythmia	44 (4.9)	30 (5.6)	9 (3.7)	2 (2.4)	3 (7.5)	0.391 †
Other comorbidities	251 (28.0)	157 (29.5)	70 (29.0)	16 (19.3)	8 (20.0)	0.162
Type of anesthesia						< 0.001 †
General	642 (73.6)	389 (75.4)	171 (73.4)	62 (74.7)	20 (50.0)	
Neuraxial	80 (9.2)	40 (7.8)	21 (9.0)	16 (19.3)	3 (7.5)	
General and neuraxial	150 (17.2)	87 (16.9)	41 (17.6)	5 (6.0)	17 (42.5)	
Total	904 (100)	539 (59.6)	241 (26.7)	84 (9.3)	40 (4.4)	

SAPS 3 - Simplified Acute Physiology Score 3; SOFA - Sequential Organ Failure Assessment Score; ICU - intensive care unit; BMI - body mass index; ASA - American Society of Anesthesiologists; CI - coronary insufficiency; COPD - chronic obstructive pulmonary disease; CRF - chronic renal failure.

* Analysis of variance;

† chi-square test.

The results are expressed as n (%) or median (interquartile range).

During the study period, 25,500 patients underwent noncardiac surgeries. Of these, 904 (3.5%, 95%CI 3.3% - 3.8%) were admitted to the ICUs and were included in the study (Figure 1).

The median (IQR) age of the patients was 62 (50 - 72) years, and 53.8% male. The median (IQR) of the SAPS 3 was 42 (32 - 53) points. Approximately half (51.7%) of the patients included in the study were treated at public ICUs. Approximately 80.4% of the patients had at least one comorbidity, with hypertension, cancer and smoking being the most frequent. Clinical and demographic characteristics and the types of surgeries performed according to geographic distribution are shown in table 1.

### Primary outcome

The 28-day postoperative mortality rate for the entire cohort was 9.6%. In the logistic regression model, the independent factors associated with 28-day mortality were age (OR = 1.032, 95% CI 1.011 - 1.052), SAPS 3 (OR = 1.041, 95%CI 1.107 - 1.279), SOFA score on ICU admission (OR = 1.175; 95%CI 1.069 - 1.292) and emergency surgery (OR = 2.509; 95%CI 1.040 - 6.051) (Table 2).

**Table 2:** Factors related to 28-day mortality after surgery

	Univariate			Multivariate		
	OR	95%CI	p value	OR	95%CI	p value
Male sex	1.105	0.658 - 1.855	0.707			
Caucasian ethnicity	0.926	0.115 - 7.492	0.943			
Age (years)	1.019	1.003 - 1.035	0.017	1.032	1.011 - 1.052	0.003
BMI (kg/cm^2^)	0.971	0.918 - 1.027	0.301			
SAPS 3 score (unit)	1.076	1.057 - 1.096	0.000	1.041	1.018 - 1.065	0.001
SOFA admission (unit)	1.281	1.198 - 1.369	0.000	1.175	1.069 - 1.292	0.001
ASA (unit)	2.326	1.684 - 3.213	0.000	1.283	0.884 - 1.863	0.190
Surgical time (minutes)	0.998	0.996 - 1.000	0.065			
Type of surgery						
Elective	Reference					
Urgent	3.577	1.880 - 6.806	0.000	1.535	0.690 - 3.414	0.294
Emergency	6.739	3.659 - 12.411	0.000	2.509	1.040 - 6.051	0.041
Surgery						
Head and neck	4.247	1.077 - 16.754	0.039	3.100	0.847 - 11.351	0.088
Abdominal	3.988	1.613 - 9.856	0.003	1.441	0.694 - 2.993	0.327
Cancer	0.39	0.434 - 1.622	0.602			
Neurological	3.754	1.331 - 10.593	0.012	1.519	0.630 - 3.664	0.352
Orthopedic	2.186	0.774 - 6.75	0.140			
Vascular	2.124	0.604 - 7.463	0.240			
Thoracic	1.176	0.195 - 7.101	0.854			
Urological	2.374	0.680 - 8.293	0.175			
Gynecological	1.325	0.151 - 11.596	0.799			
Chronic diseases						
Hypertension	1.083	0.775 - 1.514	0.639			
Cancer	1.089	0.597 - 1.920	0.774			
Diabetes mellitus	1.199	0.639 - 2.164	0.557			
Smoking	1.165	0.576 - 2.221	0.654			
Coronary insufficiency	1.010	0.365 - 2.393	0.983			
Stroke	2.689	0.689 - 9.162	0.123			
Chronic obstructive pulmonary disease	1.325	0.471 - 3.227	0.560			
Chronic renal failure	2.039	0.786 - 5.289	0.143			
Alcoholism	1.361	0.435 - 3.579	0.558			
Arrhythmia	3.007	1.181 - 7.656	0.021	0.765	0.218 - 2.686	0.676
Anemia prior to surgery (Hb <10g/dL)	1.211	0.246 - 5.954	0.814			
Other comorbidities	1.271	0.738 - 2.189	0.388			
Type of anesthesia						
General anesthesia	Reference					
Neuraxial anesthesia	0.522	0.183 - 1.489	0.224			
Combined anesthesia (general + neuraxial)	0.544	0.252 - 1.172	0.120			
Type of hospital						
Private	Reference					
Public	1.332	0.804 - 2.207	0.265			

OR - odds ratio; 95%CI - 95% confidence interval; BMI - body mass index; SAPS 3 - Simplified Acute Physiology Score 3; SOFA - Sequential Organ Failure Assessment Score; ASA - American Society of Anesthesiologists; Hb - hemoglobin level. Area under the curve: 0.843; 95% confidence interval 0.813 - 0.870.

### Secondary outcomes

**Figure 2:**
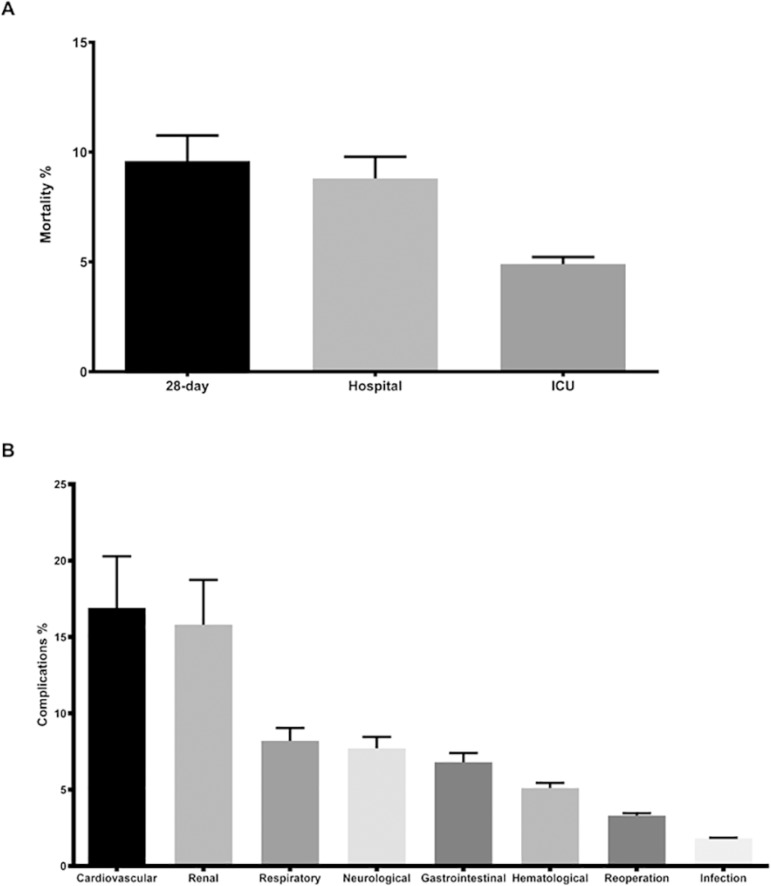
Occurrence of and confidence intervals for mortality (A) and postoperative complications (B). ICU - intensive care unit.

The total incidence of postoperative complications was 29.9% (265/886), with a higher occurrence of cardiovascular (16.9%), renal (15.8%), respiratory (8.2%) and neurological (7.7%) complications (Figure 2). The median (IQR) length of ICU stay was 2 (1 - 4) days. The median (IQR) length of hospital stay was 9.5 (5.4 - 18.6) days.

Higher SAPS 3 values (OR = 1.02; 95%CI 1.01 - 1.03) and SOFA scores on ICU admission (OR = 1.17; 95%CI 1.09 - 1.25), longer surgical times (OR = 1.001; 95%CI 1.000 - 1.002) and emergency surgeries (OR = 1.93; 95%CI 1.10 - 3.38) showed an independent association with the occurrence of complications in the ICU (Table 3).

**Table 3:** Factors related to complications in the postoperative period

	Univariate			Multivariate		
	OR	95%CI	p value	OR	95%CI	p value
Male sex	1.205	0.874 - 1.667	0.257			
Caucasian ethnicity	0.835	0.289 - 2.410	0.739			
Age (year)	1.003	0.995 - 1.012	0.458			
BMI (kg/cm^2^)	0.984	0.953 - 1.016	0.324			
SAPS 3 score (unit)	1.048	1.037 - 1.061	< 0.001	1.025	1.012 - 1.039	0.000
SOFA admission (unit)	1.249	1.189 - 1.316	< 0.001	1.172	1.095 - 1.254	0.000
ASA, unit	1.345	1.106 - 1.638	0.003	0.993	0.778 - 1.267	0.956
Surgical time (minutes)	1.002	1.000 - 1.003	0.009	1.001	1.000 - 1.002	0.012
Type of surgery						
Elective	Reference					
Urgent	2.111	1.441 - 3.092	< 0.001	1.418	0.868 - 2.315	0.163
Emergency	3.570	2.398 - 5.316	< 0.001	1.928	1.100 - 3.381	0.022
Surgery						
Head and neck	0.592	0.268 - 1.305	0.194			
Gynecological	1.503	0.576 - 3.921	0.405			
Abdominal	1.665	1.221 - 2.270	0.001	1.153	0.791 - 1.682	0.458
Vascular	1.161	0.697 - 1.936	0.566			
Thoracic	1.113	0.613 - 2.019	0.725			
Neurological	0.836	0.582 - 1.201	0.332			
Urological	0.863	0.449 - 1.658	0.658			
Cancer	1.276	0.493 - 3.599	0.627			
Orthopedic	0.796	0.531 - 1.195	0.272			
Previous chronic diseases						
Hypertension	1.083	0.775 - 1.514	0.639			
Diabetes mellitus	0.903	0.600 - 1.359	0.625			
Smoking	1.446	0.935 - 2.234	0.097			
Alcoholism	1.939	1011 - 3.718	0.046	1.171	0.557 - 2.459	0.678
Chronic renal failure	2.047	1.107 - 3.708	0.031	1.180	0.554 - 2.515	0.668
Arrhythmia	1.643	0.840 - 3.096	0.133			
Coronary insufficiency	0.933	0.524 - 1.660	0.813			
Cancer	1.011	0687 - 1.466	0.956			
Stroke	1.377	0.600 - 3.161	0.450			
Chronic obstructive pulmonary disease	0.888	0.438 - 1.676	0.726			
Anemia prior to surgery (Hb <10g/dL)	3.024	1.025 - 8.921	0.045	2.504	0.726 - 8.630	0.146
Other comorbidities	0.909	0.658 - 1.256	0.564			
Type of anesthesia						
General anesthesia	Reference					
Neuraxial anesthesia	1.681	0.632 - 4.012	0.264			
Combined anesthesia (general + neuraxial)	1.453	0.763 - 2.673	0.240			
Type of hospital						
Private	Reference			Reference		
Public	1.992	1.183 - 3.424	0.011	1.049	0.729 - 1.511	0.796

OR - odds ratio; 95%CI - 95% confidence interval; BMI - body mass index; SAPS 3 - Simplified Acute Physiology Score 3; SOFA - Sequential Organ Failure Assessment Score; ASA - American Society of Anesthesiologists; Hb: hemoglobin. Area under the curve: 0.755; confidence interval of 95% 0.722 - 0.786.

**Figure 3:**
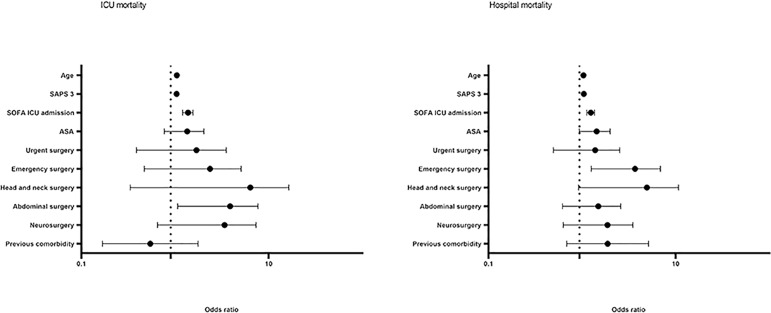
Risk factors related to intensive care unit and hospital mortality (multivariate analysis). ICU - intensive care unit; SAPS 3 - Simplified Acute Physiology Score 3; SOFA - Sequential Organ Failure Assessment Score; ASA - American Society of Anesthesiologists.

Finally, for ICU and hospital mortality, the same regression model that was performed for 28-day mortality revealed the same risk factors for hospital mortality. However, for ICU mortality, abdominal surgeries were also related to a higher risk of death (OR = 1.067; 95%CI 2.865 - 7.691) (Figure 3).

### Comparisons between public and private intensive care units

Approximately 52% of the ICUs participating in the study were public (Table S2 - Supplementary material).

In the regression model, there were no differences between public and private ICUs in either mortality or complications (Tables 2 and 3).

## DISCUSSION

In this prospective cohort study, the epidemiology, complications and mortality of high surgical risk patients in Brazil were evaluated. The main findings of the present study were a 28-day mortality rate of 9.6% and a postoperative complications rate of 30%, which the latter more frequently related to the cardiovascular and renal systems. Risk factors for complications and mortality were identified and included high SAPS 3 value and SOFA score on ICU admission, older age, prolonged surgical time and emergency surgery. A low rate of ICU admission was also observed relative to the demand for surgeries.

Data from the current study identified an ICU mortality rate of 4.9%, a hospital mortality rate of 8.9%, and a 28-day mortality rate of 9.6%. These data are similar to those of a European study (EuSOS)^(6)^ and an African study (SASOS)^(23)^ that investigated and monitored patients for at least 7 days after noncardiac surgery and reported ICU mortality rates of 3% to 5%, with a median length of ICU stay of 2 to 3 days and a median hospital stay of 9 to 10 days. In addition, when the results of the present study were compared with data from a previous Brazilian study^(8)^ in a very similar population, the morbidity and mortality rates were substantially lower, with a 15% ICU mortality rate and a 20% hospital mortality rate. These data, together with those from other large recent studies conducted in other countries^(24)^ that also found decreasing mortality and complication rates, suggest that outcomes are improving for patients with higher surgical risk, although this could be explained by nonnoticeable differences among the populations included in the studies.

However, the rate of complications in the ICU was high, especially those related to renal and cardiovascular dysfunction. Complications remain an important determinant of a short survival time, even in patients who survive hospitalization.^(2)^

Among postoperative complications, cardiovascular and renal complications are responsible for a considerable proportion of surgery-related morbidity and mortality.^(25,26)^ Factors related to perioperative care, such as fluid overload^(22,27)^ and unfavorable hemodynamic conditions, may contribute to the deterioration of cardiac and renal function.

Some characteristics are noteworthy in this sample, such as an older age of 62 (50 - 72) years, a SOFA score on ICU admission of 2 (1 - 5) and a prolonged surgical time of 240 (180 - 360) minutes. These variables, together with high SAPS 3 values and emergency surgeries, were strongly associated with 28-day mortality and postoperative complications.

Inclusion criteria previously reported in other studies,^(9,28-31)^ such as old age, clinical conditions and extensive surgeries, yielded findings similar to those of the present study.

In contrast, some prognostic scores, such as the ASA Physical Status Classification, are frequently used in clinical practice to stratify the risk of death in surgical patients; however, this score does not incorporate variables specific to the surgical procedure. Perhaps for this reason, we did not find any correlation of the ASA status with death or postoperative complications in this study. However, the SAPS 3 has gained prominence as a prognostic score in Brazilian studies of high-risk surgical patients.^(12,32)^

In addition to these assumptions, large surgeries impose physiological stresses, which can cause significant morbidity and mortality in the perioperative period.^(2,33)^ However, only 3.5% (95%CI 3.3 - 3.8) of patients undergoing major surgeries during this period were referred to the ICU. A study conducted in the United Kingdom with surgical patients reported an overall perioperative mortality rate of 2% and showed that 80% of these deaths occurred for a small subgroup of procedures that constitutes only 12% of the surgical population.^(34)^ This shows that morbidity and mortality tend to occur in a relatively small subsample of surgical patients. For this reason, it is important to identify patients at increased risk.

Our study has some strengths and limitations that we should consider, such as its multicenter design and the inclusion of ICUs with similar profiles and in proportions consistent with the regional distribution of ICUs in Brazil according to the 2016 AMIB census. Nevertheless, our study model is not robust enough to be generalized to the national level, since less than 5% of the ICUs were located in the North and Northeast Regions, and a variable degree of selection bias can result in significant differences between reports. In addition, there was a reasonable rate of refusal to participate in the study, which somewhat reduces the study’s external validity.

There were failures in capturing some relevant data that could have been included in the analyses, such as intraoperative data; however, this was not the main objective of the study as its focus was on epidemiological factors rather than on the individual care of patients.

Nonetheless, the need to obtain informed consent in epidemiological studies such as this tends to skew the sample due to the nonconsent of more severe patients in cases in which the family may be psychologically distressed. Another aspect to be considered was the lack of standardization among the centers regarding indications for postoperative intensive care. In addition, this study was not able to assess complications and mortality over long periods, and some complications may have occurred after the period analyzed in the study.

## CONCLUSION

In this sample of intensive care units in Brazil, the mortality rates of high-risk surgical patients are decreasing and are comparable to those of other regions of the world. Complications are still frequent, occurring in approximately one-third of patients. Age, SAPS 3, SOFA score on admission to the intensive care unit, emergency surgeries and surgical time were associated with 28-day mortality and postoperative complications.
