# Supplementary material for: Epidemiology and outcome of high-surgical-risk patients admitted to an intensive care unit in Brazil
Source: Rev Bras Ter Intensiva. 2020 Jan-Mar;32(1):17–27. doi: 10.5935/0103-507X.20200005 (PMC7206944; doi:10.5935/0103-507X.20200005)
Supplement: Supplementary file 1 [file rbti-32-01-0017-suppl01.pdf]

Epidemiology and outcome of high-surgical-risk patients admitted to an intensive care unit in Brazil

Epidemiologia e desfecho dos pacientes de alto risco cirúrgico admitidos em unidades de terapia intensiva no Brasil

João Manoel Silva Júnior[1,2,6], Renato Carneiro de Freitas Chaves[1,3], Thiago Domingos Corrêa[1], Murillo Santucci Cesar de Assunção[1], Henrique Tadashi Katayama[4], Fabio Eduardo Bosso[5], Cristina Prata Amendola[6], Ary Serpa Neto[1], Luiz Marcelo Sá Malbouisson[2], Neymar Elias de Oliveira[7], Viviane Cordeiro Veiga[8], Salomón Soriano Ordinola Rojas[8], Natalia Fioravante Postalli[8], Thais Kawagoe Alvarisa[8], Bruno Melo Nobrega de Lucena[2], Raphael Augusto Gomes de Oliveira[2], Luciana Coelho Sanches[6], Ulysses Vasconcellos de Andrade e Silva[6], Antonio Paulo Nassar Junior[9], Álvaro Réa-Neto[10], Alexandre Amaral[11], José Mário Teles[11], Flávio Geraldo Rezende de Freitas[12], Antônio Tonete Bafi[12], Eduardo Souza Pacheco[12], Fernando José Ramos[13], José Mauro Vieira Júnior[13], Maria Augusta Santos Rahe Pereira[14], Fábio Sartori Schwerz[14], Giovanna Padoa de Menezes[14], Danielle Dourado Magalhães[15], Cristine Pilati Pileggi Castro[15], Sabrina Frighetto Henrich[15], Diogo Oliveira Toledo[16], Bruna Fernanda Camargo Silva Parra[16], Fernando Suparregui Dias[17], Luiza Zerman[17], Fernanda Formolo[17], Marciano de Sousa Nobrega[18], Claudio Piras[19], Stéphanie de Barros Piras[19], Rodrigo Conti[19], Paulo Lisboa Bittencourt[20], Ricardo Azevedo Cruz D'Oliveira[20], André Ricardo de Oliveira Estrela[20], Mirella Cristine de Oliveira[21], Fernanda Baeumle Reese[21], Jarbas da Silva Motta Júnior[22], Bruna Martins Dzivielevski da Câmara[22], Paula Geraldes David-João[22], Luana Alves Tannous[23], Viviane Bernardes de Oliveira Chaiben[23], Lorena Macedo Araújo Miranda[24], José Arthur dos Santos Brasil[25], Rafael Alexandre de Oliveira Deucher[10], Marcos Henrique Borges Ferreira[26], Denner Luiz Vilela[26], Guilherme Cincinato de Almeida[26], Wagner Luis Nedel[27], Matheus Golenia dos Passos[27], Luiz Gustavo Marin[27], Wilson de Oliveira Filho[28], Raoni Machado Coutinho[28], Michele Cristina Lima de Oliveira[28], Gilberto Friedman[29], André Meregalli[29], Jorge Amilton Höher[29], Afonso José Celente Soares[30], Suzana Margareth Ajeje Lobo[7]

Table S1 - Profile of the centers included in the study according to geographic distribution

Characteristics	All	Southeast	South	Central-West	North and Northeast	p value
Number of hospital beds	303 (213 - 646.5)	450 (227.5 - 831.7)	222 (139.2 - 429.7)	407 (311.7 - 601.2)	281 (259 - 303)	0.504*
Type of patients treated at the ICU						0.326†
Only surgical	4 (13.3)	3 (20)	0 (0)	1 (33.3)	0 (0)	
Surgical and clinical	26 (86.7)	12 (80)	10 (100)	2 (66.7)	2 (100)	
Number of ICU beds	36 (20.2 - 56.5)	32 (22.5 - 52.2)	35 (21.5 - 50)	57 (24.7 - 84)	30.5 (21 - 40)	0.922*
Hospitals with semi-intensive care	9 (30)	6 (40)	2 (20)	0 (0)	1 (50)	0.414*
Centers with ICU residency	20 (66.7)	10 (66.7)	5 (50)	3 (100)	2 (100)	0.290*
Centers with general surgery residency	20 (66.7)	11 (73.3)	5 (50)	3 (100)	1(50)	0.348*
ICUs with physical therapy available 24 hours a day	20 (74.1)	11 (73.3)	7 (87.5)	1 (50)	1 (50)	0.580*
Number of physical therapists per patient	10 (8.0 - 10.0)	10 (9.2 - 10)	10 (10 - 10)	7.5 (7 - 8)	10 (10 - 10)	0.24*
Number of nurses per patient	6.5 (4.0 - 10.0)	7 (4 - 10)	10 (10 - 10)	5 (4 - 6)	7 (4 - 10)	0.613*
Number of physicians per patient	10 (7.0 - 10.0)	10 (6.7 - 10)	10 (10 - 10)	8 (8 - 8)	8 (6 - 10)	0.688*
Type of ICU						0.308†
Public	15 (51.7)	5 (35.7)	6 (75)	3 (75)	1 (33.3)	
Private	14 (48.3)	9 (64.2)	2 (25)	1 (25)	2 (66.7)	

ICU - intensive care unit. * Analysis of variance; † chi-square test. The results are expressed as median (interquartile range) or n (%).

Table S2 - Comparison of public and private intensive care units

Characteristics	All	Public	Private	p value *
Logistics of ICU operation				
Number of hospital beds	303 (216.5 - 636.75)	303 (226.5 - 742.5)	309.5 (120.0 - 627.0)	0.7434
Type of patients treated in the ICU				0.283
Only surgical	4 (13.3)	3 (20)	1 (6.7)	
Surgical and clinical	26 (86.7)	12 (80)	14 (93.3)	
Number of ICU beds	36 (20.5 - 56)	40 (20 - 57)	30 (21 - 55)	0.8360
Hospitals with semi-intensive care	9 (30)	3 (20)	6 (40)	0.232
Centers with ICU residency	20 (66.2)	11 (73.3)	9 (60)	0.439
Centers with general surgery residency	20 (66.7)	13 (86.7)	7 (46.7)	0.020

Continue...

... continuation

Characteristics	All	Public	Private	p value *
ICUs with physical therapy available 24 hours a day	20 (74.1)	8 (66.7)	12 (80)	0.432
Number of physical therapists per patient	10 (8.0 - 10.0)	10 (10 - 10)	10 (7 - 10)	0.2573
Number of nurses per patient	6.5 (4.0 - 10.0)	10 (5.5 - 10)	6 (4.0 - 9.25)	0.2616
Number of physicians per patient	10 (7.0 - 10)	10 (6.25 - 10)	10 (7.25 - 10)	0.5694
Patient characteristics				
n total patients (%)	904 (100)	468 (51.7)	436 (48.2)	0.484†
Age, years	62 (50 - 72)	63 (52 - 73)	62 (49 - 72)	0.686†
Men	444 (53.8)	236 (55.9)	208 (51.6)	0.241†
SAPS 3	42 (32 - 53)	45 (34 - 56)	40 (31 - 50)	< 0.001†
SOFA	2 (1 - 5)	3 (1 - 5)	1 (0 - 4)	< 0.001†
BMI	25 (22 - 28)	25 (22 - 28)	25 (22 - 28)	0.773†
Ethnicity				
Caucasian	585 (71.3)	314 (71.0)	271 (71.7)	0.898*
Brown	157 (19.1)	74 (16.7)	83 (22.0)	0.071*
Black	61 (7.4)	42 (9.5)	19 (5.0)	0.021*
Other	17 (2.1)	12 (2.8)	2 (1.3)	1.000*
ASA	2 (2 - 3)	3 (2 - 3)	2 (2 - 3)	< 0.001†
Duration of surgery, minutes	240 (180 - 360)	240 (180 - 360)	260 (180 - 390)	< 0.001†
Types of surgery				
Elective	613 (69.2)	273 (59.2)	340 (80.0)	< 0.001*
Urgent	147 (16.6)	85 (18.4)	62 (14.6)	< 0.081*
Emergency	126 (14.2)	93 (20.2)	33 (7.8)	< 0.001*
Surgery				
Abdominal	252 (28.1)	115 (25.0)	137 (31.4)	0.039*
Cancer	250 (27.9)	90 (19.6)	160 (36.7)	< 0.001*
Neurological	186 (20.8)	68 (14.8)	118 (27.1)	< 0.001*
Orthopedic	143 (16.0)	78 (17.0)	65 (14.9)	0.456*
Vascular	74 (8.3)	61 (13.3)	13 (3.0)	< 0.001*
Thoracic	53 (5.9)	23 (5.0)	30 (6.9)	0.293*
Urological	23 (4.8)	14 (5.7)	9 (4.0)	0.530*
Head and neck	39 (4.4)	19 (4.1)	20 (4.6)	0.864*
Gynecological	16 (3.4)	7 (3.1)	9 (3.6)	0.948*
Other	55 (6.1)	34 (7.4)	21 (4.8)	0.180*
Underlying disease	707 (80.4)	354 (78.8)	353 (82.1)	< 0.001*
Hypertension	396 (44.2)	211 (45.9)	185 (42.4)	0.003*
Cancer	191 (21.3)	55 (12.0)	136 (31.2)	< 0.001*
Diabetes mellitus	188 (21.0)	105 (22.8)	83 (19.0)	0.002*
Smoking	134 (15.0)	97 (21.1)	37 (8.5)	0.251*
Coronary insufficiency	67 (7.5)	36 (7.8)	31 (7.1)	< 0.001*
COPD	54 (6.0)	30 (6.5)	24 (5.5)	0.520*
Chronic renal failure	48 (5.4)	26 (5.7)	22 (5.0)	0.002*
Stroke	27 (3.0)	20 (4.3)	7 (1.6)	0.700*
Alcoholism	46 (5.1)	33 (7.2)	13 (3.0)	0.241*
Arrhythmia	44 (4.9)	21 (4.6)	23 (5.3)	0.391*
Type of anesthesia				
General anesthesia	642 (73.6)	314 (70.7)	328 (76.6)	0.006*
Neuraxial anesthesia	80 (9.2)	49 (11.0)	31 (7.2)	0.009*
General and neuraxial anesthesia	150 (17.2)	81 (18.2)	69 (16.1)	< 0.001*

ICU - intensive care unit; SAPS 3 - Simplified Acute Physiology Score 3; SOFA - Sequential Organ Failure Assessment Score; BMI - body mass index; ASA - American Society of Anesthesiologists; COPD - chronic obstructive pulmonary disease. * analysis of variance; † chi-square. The results are expressed as the median (interquartile range) or n (%).
